# Temperature-Dependent Alkyl Glycerol Ether Lipid Composition of Mesophilic and Thermophilic Sulfate-Reducing Bacteria

**DOI:** 10.3389/fmicb.2017.01532

**Published:** 2017-08-09

**Authors:** Arnauld Vinçon-Laugier, Cristiana Cravo-Laureau, Isabelle Mitteau, Vincent Grossi

**Affiliations:** ^1^Laboratoire de Géologie de Lyon, UMR CNRS 5276, Université Lyon 1 Villeurbanne, France; ^2^Equipe Environnement et Microbiologie, UMR CNRS 5254, Université de Pau et des Pays de l’Adour, Institut des Sciences Analytiques et de Physico-Chimie pour l’Environnement et les Matériaux Pau, France

**Keywords:** bacterial ether lipids, membrane fluidity, branched-chain alkyl glycerols, homeoviscous adaptation, environmental proxies

## Abstract

The occurrence of non-isoprenoid alkyl glycerol ether lipids in Bacteria and natural environments is increasingly being reported and the specificity and diagenetic stability of these lipids make them powerful biomarkers for biogeochemical and environmental studies. Yet the environmental controls on the biosynthesis of these peculiar membrane lipids remain poorly documented. Here, the lipid content of two mesophilic (*Desulfatibacillum aliphaticivorans* and *Desulfatibacillum alkenivorans*) and one thermophilic (*Thermodesulfobacterium commune*) sulfate-reducing bacteria—whose membranes are mostly composed of ether lipids—was investigated as a function of growth temperature (20–40°C and 54–84°C, respectively). For all strains, the cellular lipid content was lower at sub- or supra-optimal growth temperature, but the relative proportions of dialkyl glycerols, monoalkyl glycerols and fatty acids remained remarkably stable whatever the growth temperature. Rather than changing the proportions of the different lipid classes, the three strains responded to temperature changes by modifying the average structural composition of the alkyl and acyl chains constitutive of their membrane lipids. Major adaptive mechanisms concerned modifications of the level of branching and of the proportions of the different methyl branched lipids. Specifically, an increase in temperature induced mesophilic strains to produce less dimethyl branched dialkyl glycerols and 10-methyl branched lipids relative to linear structures, and the thermophilic strain to decrease the proportion of *anteiso* relative to *iso* methyl branched compounds. These modifications were in agreement with a regulation of the membrane fluidity. In one mesophilic and the thermophilic strains, a modification of the growth temperature further induced changes in the relative proportions of *sn*-2 vs *sn*-1 monoalkyl glycerols, suggesting an unprecedented mechanism of homeoviscous adaptation in Bacteria. Strong linear correlations observed between different ratios of alkyl glycerols and temperature allow to hypothesize the use of these specific lipids as indicators of temperature changes in the environment.

## Introduction

The physiological functions of cellular membranes may be strongly influenced by fluctuations of environmental parameters such as temperature, salinity, pH, or hydrostatic pressure (Russell et al., 1995). In response to changing environmental conditions, prokaryotes modify the composition of their membrane to maintain optimal membrane properties (Zhang and Rock, 2008). Pioneer studies devoted to the adaptation of bacterial membranes were performed using *Escherichia coli* grown at different temperatures. The strain was shown to modify its lipid composition according to growth temperature without changing the physical properties of its membrane (Marr and Ingraham, 1962; Sinensky, 1971). These observations gave rise to the theory of homeoviscous adaptation (Sinensky, 1974). Since then, the influence of temperature on bacterial membranes has been intensively studied and is now relatively well constrained in terms of physiological and physicochemical adaptation (Ernst et al., 2016 and references therein). Temperature is known to influence the membrane fluidity by altering the lipid ordering, the lateral and rotational diffusion of proteins and the resistance of the membrane to shear forces (Sperotto et al., 1989). Bacteria grow at a temperature corresponding to or just above the phase transition temperature at which membrane lipids are in a liquid crystalline state (Mykytczuk et al., 2007). This phase transition temperature strongly depends on the chemical structure of membrane lipids.

Bacterial membrane lipids generally consist of fatty acids (FAs) linked via ester bonds to the *sn*-1 and *sn*-2 carbons of a *sn*-glycerol-3 backbone (so-called acyl glycerols), the third carbon of the glycerol moiety being attached to a phospho- or glyco-polar head group (Lechevalier and Moss, 1977; Zhang and Rock, 2008). To control the phase transition temperature of membrane lipids and maintain adequate membrane properties in response to changes in temperature, bacteria adjust the FA composition and, to a lesser extent, the nature of the head group of their phospholipids (Russell, 1984; Ernst et al., 2016). The main adaptive mechanisms concern modifications of the chain length and degree of unsaturation (adding/removal of double bonds or rings) of the acyl chains and of the proportion of branched (classically *iso*/*anteiso*) FAs (Russell, 1984, 1989; Denich et al., 2003; Koga, 2012).

In addition to acyl glycerols, some bacteria also synthesize alkyl glycerol ether lipids (AGEs; Langworthy et al., 1983; Grossi et al., 2015 and references therein). These less common membrane lipids consist of non-isoprenoid alkyl chains linked to the *sn*-1 and *sn*-2 carbons of a *sn*-3 glycerol by ether bridges instead of the ester bounds found in acyl glycerols. AGEs have often been considered as a characteristic of (hyper)thermophilic bacteria due to their chemical resistance relative to ester linkages and their systematic occurrence in deep-branching thermophilic bacterial lineages (De Rosa et al., 1974; Langworthy et al., 1983; Pond and Langworthy, 1986; Huber et al., 1992; Zeng et al., 1992). However, the presence of such lipids in diverse mesophilic bacteria (Caillon et al., 1983; Asselineau and Asselineau, 1990; Rütters et al., 2001; Sinninghe Damsté et al., 2011; Grossi et al., 2015; Vinçon-Laugier et al., 2016) and non-extreme environments (Schouten et al., 2000; Sinninghe Damsté et al., 2000; Pancost et al., 2001; Schubotz et al., 2009; Hernandez-Sanchez et al., 2014) argue against this conceptual view. In fact, since their discovery, an increasing variety of AGEs has been reported from pure strains of Bacteria and diverse natural settings covering a large range of environmental conditions (Grossi et al., 2015; Vinçon-Laugier et al., 2016 and references therein). The main structures of AGEs reported so far are 1-*O*-monoalkyl (1-*O*-MAGEs) and 2-*O*-monoalkyl (2-*O*-MAGEs) glycerol monoethers (containing an eventual acyl chain at the *sn*-2 or *sn*-1 position of the glycerol, respectively), 1,2-*O*-dialkyl glycerol diethers (DAGEs), branched glycerol dialkyl glycerol tetraethers (brGDGTs) and plasmalogens (1-alk-1′-enyl glycerols with an acyl chain at the *sn*-2 position of the glycerol). Except plasmalogens which are widespread in Eukarya and Bacteria, non-isoprenoid ether lipids appear specific to Bacteria. MAGEs are the most common structures and, until now, have been reported in 16 families of Bacteria from eight different phyla (Grossi et al., 2015; Vinçon-Laugier et al., 2016 and references therein). DAGEs have been reported from a more restricted number of bacterial families including the Cystobacteraceae (Caillon et al., 1983; Asselineau and Asselineau, 1990), the Desulfobacteraceae (Grossi et al., 2015; Vinçon-Laugier et al., 2016), the Planctomycetaceae (Sinninghe Damsté et al., 2005), the Thermoanaerobacteraceae (Huber et al., 1996), the Aquificaceae (Huber et al., 1992), and the Thermodesulfobacteriaceae (Langworthy et al., 1983; Sturt et al., 2004; Hamilton-Brehm et al., 2013). To date, brGDGTs have only been observed in some Thermotogaceae (Sinninghe Damsté et al., 2007) and in two species of Acidobacteriaceae (Sinninghe Damsté et al., 2011). Logically, MAGEs are systematically present in bacteria able to synthesize DAGEs or brGDGTs.

Due to their widespread occurrence in the environment and their diagenetic stability, bacterial ether lipids are often considered as powerful biomarkers. During the last decade, different empirical studies based on terrestrial settings have shown that the structural distribution of brGDGTs varies with the mean annual air temperature and the pH of soils (Schouten et al., 2000; Weijers et al., 2007). Consequently, based on extensive surface soil calibrations (Weijers et al., 2007; Peterse et al., 2012), brGDGTs have been intensively used as molecular proxies to reconstruct past variations in mean air temperature and soil pH, although uncertainties remain on their biological source and the physiological parameters controlling their distribution (Schouten et al., 2013 and references therein). Some attempts have also been made to link variations in AGE structural distribution to environmental variables such as temperature and pH (Hernandez-Sanchez et al., 2014; Kaur et al., 2015; Yang et al., 2015). The usefulness of AGEs as environmental proxies appears, however, strongly limited by the lack of information available on the physiological response of AGE-containing membranes (and especially on the precise structural modifications incurred by AGEs) in response to changing environmental conditions. Indeed, few studies have been dedicated to the modification of the ether lipid composition of isolated bacterial strains in response to modifications of their growth conditions (Sinninghe Damsté et al., 2014). Such studies, performed under controlled laboratory conditions, should allow for a better understanding of the mechanisms involved in homeoviscous adaptation of ether-containing bacterial membranes and, in turn, may improve the use or allow the development of environmental molecular indicators based on these peculiar membrane lipids.

We recently reported that the AGE composition of mesophilic heterotrophic sulfate-reducing bacteria (SRB, from the genus *Desulfatibacillum*) strongly depends on the nature and the chain length of the carbon substrate used for growth (Vinçon-Laugier et al., 2016). However, whereas the number of MAGE and DAGE homologs synthesized from a single substrate may vary from few to more than 50, the average chain length (ACL) and level of branching of MAGEs and DAGEs were shown to remain remarkably stable for all substrates, illustrating a compositional control of ether lipids to maintain optimal membrane properties in response to changing growth substrate (Vinçon-Laugier et al., 2016). Here, we investigated the influence of growth temperature on the qualitative and quantitative ether lipid composition of these mesophilic SRB grown (on a single substrate) between 20 and 40°C. Comparison with a thermophilic strain (*Thermodesulfobacterium commune*) grown between 54 and 84°C allowed characterizing typical changes in the chemical composition of ether-containing bacterial membranes in relation (or not) to temperature physiological preferences.

## Materials and Methods

### Source of Bacteria and Culture Conditions

*Desulfatibacillum aliphaticivorans* strain CV2803^T^ and *Desulfatibacillum alkenivorans* strain PF2803^T^ are mesophilic SRB belonging to the family Desulfobacteraceae within the class Deltaproteobacteria, whose membranes lipids have been shown to contain significant proportions of MAGEs and DAGEs (Grossi et al., 2015; Vinçon-Laugier et al., 2016). Both strains were isolated from oil-polluted sediments and show some distinctive physiological traits (Cravo-Laureau et al., 2004a,b). The present consideration of both strains allowed comparing the response to changing growth conditions between species of the same genus. The thermophilic SRB *T. commune* strain DSM 2178^T^ belonging to the family Thermodesulfobacteriaceae within the class Thermodesulfobacteria was selected for this study due to its capacity to biosynthesize MAGEs and DAGEs with certain structural similarities and clear distinctions compared to AGEs biosynthesized by the mesophilic strains (Langworthy et al., 1983; Sturt et al., 2004). Strain DSM 2178 was isolated from Inkpot Spring at Yellowstone National Park and described by Zeikus et al. (1983).

*Desulfatibacillum* strains were cultivated on synthetic sulfate-reducing medium (Cravo-Laureau et al., 2004a,b) with octanoate (>99%, Sigma–Aldrich) as sole source of carbon and energy. *T. commune* was grown in medium 206 (DSMZ) with lactate (>99%, Sigma–Aldrich) as sole source of carbon and energy (Langworthy et al., 1983). Growth substrates were chosen to allow for a rapid growth and the production of a wide diversity of AGEs (Langworthy et al., 1983; Vinçon-Laugier et al., 2016). All cultures were incubated under optimal NaCl concentration and pH conditions (**Table 1**) whereas the growth temperature was set to optimal, sub-optimal, or supra-optimal values according to the growth interval of each strain (**Table 1**). The time of incubation required to reach the end of the exponential growth phase was adjusted based on independent kinetics of growth performed for each strain and each temperature tested. It lasted from 4 to 8 days for *Desulfatibacillum* strains and from 1 to less than 3 days for *T. commune*.

**Table 1:** Studied sulfate-reducing bacteria and growth conditions.

Species		Reference	Temperature range (°C)	Temperatures tested (°C)	[NaCl] (g/L)	pH
*Desulfatibacillum aliphaticivorans*	CV2803^T^	Cravo-Laureau et al. (2004a)	15–40	20–25–**30**–35–40	24	7.5
*Desulfatibacillum alkenivorans*	PF2803^T^	Cravo-Laureau et al. (2004b)	22–40	24**–30**–35–40	10	6.8
*Thermodesulfobacterium commune*	DSM 2178^T^	Zeikus et al. (1983)	50–85	54–60–**70**–80–84	0	7

*Optimal growth temperatures are bolded.*

Triplicate cultures were grown for each temperature tested, and each culture was sub-cultured three times under the same conditions before analysis. By the end of the exponential growth phase of the third sub-culture, a known aliquot of each culture was centrifuged and the protein concentration was determined by using the QuantiPro BCA assay kit (Sigma) with bovine serum albumin as a standard. The remaining culture was filtered on glass microfiber filters (GF/F; Whatman) which were kept frozen until lipid analysis. Eventual fractionation between small and large cells that could have occurred during filtration was checked by microscopy observations and optical density (OD) measurements of filtrates, confirming that smaller cells were also retained on the filters.

### Cell Hydrolysis

Filtered cells were hydrolyzed by refluxing for 2 h in 1 N HCl in methanol (MeOH). After cooling, the hydrolysate was adjusted to pH 4 with 2 N KOH in MeOH–water (1:1, v/v) and, following the addition of water (final H_2_O–MeOH ratio 1:1, v/v), extracted four times with dichloromethane. The combined extracts were dried over anhydrous Na_2_SO_4_, concentrated with a rotary evaporator and evaporated to dryness under a gentle stream of N_2_.

A known amount of *n*-tricosanol (*n*-C_23_-1-ol) used as an internal standard was added to each hydrolyzed extract. AGEs and the internal standard were silylated by reaction with *N,O*-bis(trimethylsilyl)trifluoroacetamide in pyridine (1:1 v/v, 50°C, 45 min) before gas chromatography-mass spectrometry (GC-MS) analysis. FAs were trans-esterified during the hydrolysis and analyzed as FAs methyl esters. The analysis of blanks, elaborated by filtering non-inoculated medium on GF/F filters, showed that lipid contamination from the filters and the medium was negligible.

### Gas Chromatography-Mass Spectrometry

GC-MS analyses were performed using an Agilent 6890N gas chromatograph interfaced to an Agilent 5975C mass spectrometer. The GC instrument was equipped with a HP5MS column (30 m × 0.25 mm × 0.25 μm) and a cool on column injector. The temperature of the inlet was programmed from 60°C (held 0.5 min) to 300°C (held 1 min) at 200°C min^-1^. The samples were injected at 60°C and the oven temperature was programmed to 130°C at 20°C min^-1^, then to 250°C at 5°C min^-1^ and finally to 300°C (held 30 min) at 3°C min^-1^. Helium was the carrier gas. The temperature of the interface line, the source and the quadrupole was 280, 200, and 150°C, respectively. The mass spectrometer was operated at 70 eV over the range of *m/z* 50–800.

### Determination of Structural Indices

For each culture analyzed, the weighted ACL of each class of lipids was calculated using Eq. 1. ACL values of AGEs were calculated without considering the glycerol moiety and eventual methyl substituents.

(1) $$\begin{array}{c} \mathrm{ACL}\;(i)=\frac{\sum_{n}\left(i\;\mathrm{with}\;C_{n}\times C_{n}\right)}{\sum_{n}i\;\mathrm{with}\;C_{n}} \end{array}$$

where i = relative abundance of total FAs, MAGEs, or DAGEs with C*_n_*, and C*_n_* = number of carbons in the alkyl/acyl chain(s). For DAGEs, C*_n_* was obtained by dividing the sum of both alkyl chain lengths by a factor 2.

Different ratios based on branched AGEs were calculated and were expressed in logarithm. Ratios based on specific branched DAGEs excluded compounds that could only be tentatively identified (i.e., DAGEs with two methyl branches at different positions). The significance of differences observed between various growth conditions was determined using a Student’s *t*-test (*p*-value). The lower and upper boundaries of the 95% (symmetric) confidence interval associated with each proportion or ratio were estimated based on the ±1.96 standard deviation between three independent cultures.

## Results and Discussion

### Lipid Composition of SRB under Optimal Growth Conditions

The hydrolyzed lipids of *Desulfatibacillum* strains grown on octanoate under optimal conditions mainly consist of 13 FAs, 15 MAGEs, and 21 DAGEs with chain length ranging from C_13_ to C_16_ (C_13_–C_18_ for FAs) and with a possible methyl branch at C-10 (10Me), *iso* (*i*), or *anteiso* (*ai*) position. The position of methyl branching in MAGEs and monomethyl branched DAGEs could be inferred from the retention order of the compounds (Vinçon-Laugier et al., 2016), but the assignment of the position of the branched alkyl chain(s) on the glycerol moiety (*sn*-1 vs *sn*-2) in DAGEs remained tentative. In both strains, FAs, MAGEs, and DAGEs accounted for ca. 40, 10, and 50% of total hydrolyzed lipids, respectively. It is noteworthy, however, that FAs were present in living cells as monoalkyl/monacyl glycerols (AAGs) and diacylglycerols leading, after hydrolysis, to an overestimation of the FA-based lipid pool relative to ether lipids.

Lipids of *T. commune* strain DSM 2178^T^ grown on lactate under optimal conditions were composed of ca. 30% of FAs, 10% of MAGEs, and 60% of DAGEs although, as mentioned for the mesophilic strains, the proportion of FA-based lipids relative to ether lipids is likely to be overestimated. Sixteen FAs ranging from C_13_ to C_20_, and 21 MAGEs and 31 DAGEs ranging from C_14_ to C_20_ were identified (Supplementary Table S1). The three classes of compounds contained linear, *iso*- or *anteiso*-branched alkyl chains. The position of methyl branching in branched MAGEs, monomethyl branched DAGEs and DAGEs with two identical branched chains (*ai*/*ai, i*/*i*) could be inferred from the retention order of the compounds. As for the mesophilic strains, the assignment of the position on the glycerol moiety (*sn*-1 vs *sn*-2) of the branched alkyl chain(s) in monomethyl branched DAGEs and DAGEs with two methyl branches at different positions (*ai*/*i, i*/a*i*) remained tentative (Supplementary Table S1). The present lipid composition of *T. commune* strain DSM 2178^T^ partly differs from that reported originally for the same strain by Langworthy et al. (1983). In addition to the identification of a higher diversity of MAGEs and DAGEs in our cultures (e.g., *iso* and *anteiso* lipids with chain lengths >C_18_; Supplementary Table S1), the relative distribution of the main homologs was significantly different between both studies. Such differences in the lipid composition of strain DSM 2178^T^ observed between its original description and the present work performed more than 30 years later can be attributed to improved gas chromatographic separation and mass spectrometric identification of the various AGE isomers synthesized by the strain, different culture conditions (yet lactate was used as growth substrate in both cases) and/or a physiological evolution of the strain over time.

Although, under optimal growth conditions, *T. commune* appeared to contain a slightly higher proportion of DAGEs than the two mesophiles (ca. 60 vs 50% of total lipids, respectively), the resemblance in lipid composition between the three strains investigated indicates that the thermal stability of lipids synthesized by bacteria is not directly related to the heat tolerance of the strain (Koga, 2012).

### Influence of Growth Temperature on the Lipid Composition of SRB

#### Total Lipids and Lipid Classes

**FIGURE 1:**
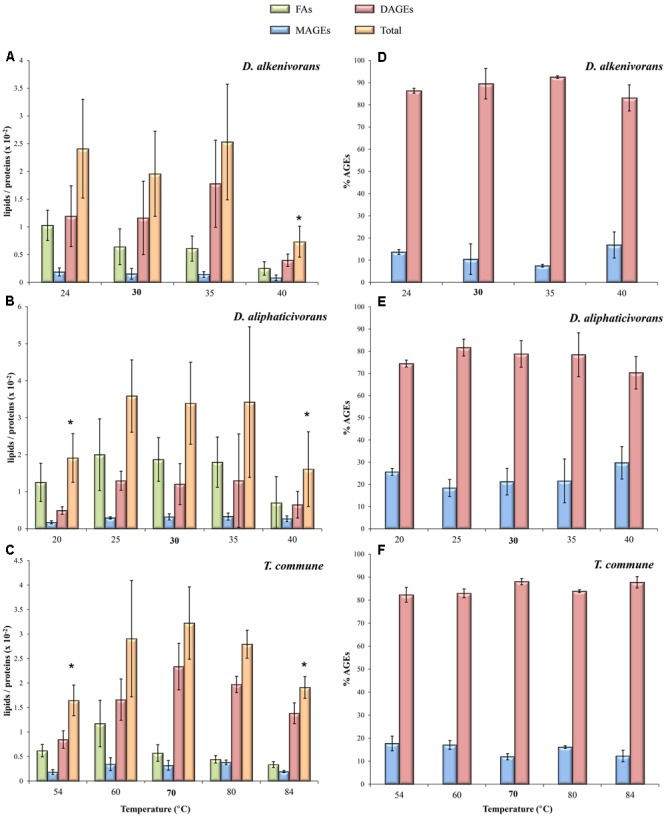
Hydrolyzed lipid content **(A–C)** and relative abundances of MAGEs and DAGEs **(D–F)** in the mesophiles *D. alkenivorans* and *D. aliphaticivorans* and the thermophile *T. commune* grown at different temperatures. Data are means of three independent cultures. Optimal growth temperatures are bolded. The asterisks indicate significant differences compared to optimal temperature as determined by the Student’s *t*-test (*p*-value < 0.05).

The cellular lipid content (relative to proteins) of the three strains appeared maximum at or around the optimal growth temperature, but showed slight changes in response to changing growth temperature, especially for growth at the limits of the temperature ranges (**Figures 1A–C**). In most cases, an increase or decrease in temperature induced a decrease of the amount of lipids relative to proteins (**Figures 1A–C**). This could be due either to a decrease of the lipid biosynthesis or to an increase of the protein content under non-optimal conditions of growth. The exact mechanism involved in the modification of this ratio in the present three strains remains unexplored and may vary from species to species. But it is possible that the temperatures differing the most from optimal conditions induced a reduction of the gene expression and/or of the activity of certain enzymes involved in lipid biosynthesis, thus limiting lipid production. An increase of the cellular protein content relative to the lipid content under non-optimal conditions appears less likely. Indeed, for the two mesophilic strains, the ratio proteins vs OD showed little variations whatever the temperature investigated (data not shown). The same ratio could not be determined for the thermophilic strain due to the presence of iron sulfide precipitates in the cultures. Moreover, cells were always analyzed at the end of the exponential phase, i.e., in comparable physiological state, likely limiting potential variations in the protein content under non-optimal conditions.

Despite these noticeable variations in the lipid to protein ratio, the relative proportion of each lipid class remained relatively stable in each strain, none being preferentially affected by shifts in temperature (**Figures 1A–C**). Remarkably, MAGEs and DAGEs accounted for ca. 20 and 80% of total AGEs, respectively, whatever the strain and the growth temperature tested (**Figures 1D–F**). The slight decrease in DAGE and related increase in MAGE proportions observed for the mesophilic *Desulfatibacillum* strains under sub- and supra-optimal conditions (**Figures 1D,E**) were not significant (*p*-value > 0.05).

Due to the thermal stability of ether bonds relative to ester bonds and to the quasi systematic occurrence of non-isoprenoid alkyl glycerol lipids in thermophilic strains, the occurrence of such compounds has often been regarded as an adaptation of Bacteria to cope with high temperatures (Sprott et al., 1991; Koga, 2012). However, not all of the ether-producing bacteria are themselves thermophilic (Grossi et al., 2015) and some thermophilic bacteria do not synthesize ether lipids (Sinninghe Damsté et al., 2007). The constant ether lipid content of the three strains investigated across their full ranges of growth temperature further argues against ether lipids constituting a direct phylogenetic adaptive strategy to thermophily (Boucher, 2007; Valentine, 2007; Koga, 2012). This, however, does not rule out an implication of these peculiar lipids in bacterial membrane adaptation to changing temperature as shown below.

#### Structural Modifications of Alkyl and Acyl Chains

Rather than changing the proportions of the different lipid classes, the three strains responded to changing growth temperature by modifying the average structural composition of their membrane lipids. These changes specifically concerned the chain length and branching pattern of the alkyl and acyl chains constitutive of the membrane lipids, as well as the position of the alkyl chain on the glycerol backbone of MAGEs (1-*O*-MAGEs vs 2-*O*-MAGEs). Because we did not analyze phospholipids in their intact form, we could not determine potential modifications of the polar head groups that might have appeared in addition to those observed for the core lipids.

##### Carbon chain length

The modification of the carbon chain length of phospholipid FAs (PLFAs) is a classical way for bacterial membranes to adapt to changing growth conditions. Generally, the ACL of PLFAs increases when the temperature increases, and vice versa (Russell, 1984). FAs with longer carbon chains have higher melting points and allow PLFAs to pack more tightly, reducing the membrane fluidity and making the membrane more gel-like in response to an increase in temperature (Quinn, 1981; Russell, 1989; Denich et al., 2003; Mykytczuk et al., 2007). Conversely, a decrease of the growth temperature classically leads to an increased proportion of FAs with shorter chains whose lower melting-points increase the membrane fluidity and keep the membrane phase transition temperature above ambient temperature (Suutari and Laakso, 1994; Denich et al., 2003; Mansilla et al., 2004; Mykytczuk et al., 2007).

**FIGURE 2:**
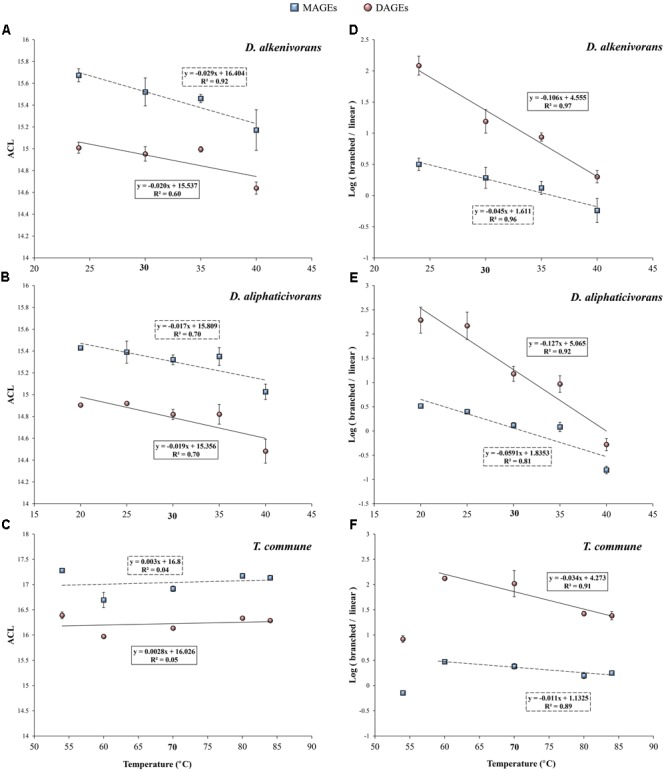
Weighted average chain length (ACL) **(A–C)** and logarithm of the ratio of branched to linear structures **(D–F)** of MAGEs and DAGEs formed by the mesophiles *D. alkenivorans* and *D. aliphaticivorans* and the thermophile *T. commune* as a function of growth temperature. Branched DAGEs correspond to all compounds with one or two branched alkyl chain(s) whereas linear DAGEs correspond to unbranched compounds (see Supplementary Table S1 and Figure S2). Each data point is the mean of three independent cultures. Optimal growth temperatures are bolded.

There is, however, a strong variability of response among Bacteria, and the modification of the ACL of membrane lipids induced by changing growth temperature often appears species-dependent. For example, Freese et al. (2008) previously reported that temperature-induced changes in the FA composition of 24 bacterial strains (representing nine genera from six phylogenetic groups) could vary from one species to another. In this case, the ACL of PLFAs showed no systematic behavior in response to changes in temperature from 10 to 50°C, sometimes being positively or negatively correlated, but most of the time remaining unchanged (Freese et al., 2008). Another study conducted with the thermophilic bacterium *Bacillus acidocaldarius* also demonstrated constant ACL values (∼15.9) of PLFAs across a higher (50–70°C) temperature range (De Rosa et al., 1974). Thus, for both mesophilic and thermophilic bacteria, other structural changes of PLFAs than modification of their carbon chain length are likely to be involved in thermal adaptation. This is the case for the present three strains. Indeed, the ACL of MAGEs, DAGEs, and FAs produced by the mesophilic *Desulfatibacillum* strains slightly decreased with increasing temperature (**Figures 2A,B** and Supplementary Figure S1A), whereas *T. commune* did not significantly modify the chain length of its membrane lipids in response to changing growth temperature (**Figure 2C** and Supplementary Figure S1A). ACL values of AGEs produced by the two mesophiles even appeared linearly correlated with temperature (0.60 < *R*^2^ < 0.92), with comparable linear regression slopes observed for a given class of compounds in both strains. Such trends in the ACL modification of membrane acyl and alkyl lipids are not consistent with the aforementioned mechanisms classically involved in homeoviscous adaptation to temperature and, therefore, suggest the existence of alternative and compensatory mechanisms. Those can consist in modifications of the degree of unsaturation (addition/removal of double bonds) and/or the level (proportion) and pattern (position) of methyl branching or cyclization (e.g., cyclopropyl rings) which have greater fluidizing effect than chain length modifications (De Rosa et al., 1974; Russell and Fukunaga, 1990; Freese et al., 2008; Knothe and Dunn, 2009). None of the present strains synthesized unsaturated or cyclic FAs and AGEs, but all three produced different types of methyl-branched alkyl and acyl chains which were thus further investigated.

It should be noted, however, that the ACL of the alkyl and acyl chains synthesized by the thermophilic strain (∼16–17) was systematically higher than that of lipids synthesized by the two mesophilic strains (∼15) (**Figures 2A–C**). This agrees with the fact that thermophilic bacteria generally have to cope with higher temperatures and thus synthesize lipids with longer carbon chains and higher melting points (Shen et al., 1970; Chan et al., 1971; Russell and Fukunaga, 1990). In line with this theory, cultures of Aquificales grown at 85°C (Jahnke et al., 2001) synthesized AGEs with a higher ACL (∼18–19) than that of *T. commune*.

##### Methyl branching

Changes in the proportions of methyl branched PLFAs are known to affect the fluidity of bacterial cytoplasmic membranes (Denich et al., 2003). Branched PLFAs restrict the ability to slide past acyl chains and form crystalline structures, thus increasing the fluidity of the membrane (Russell and Fukunaga, 1990). The proportions of branched MAGEs, DAGEs, and FAs synthesized by the present SRB strains were indeed observed to vary as a function of growth temperature (**Figure 2** and Supplementary Figures S1–S3). For the two mesophiles, the ratio of total branched vs total linear homologs appeared negatively correlated to temperature for any class of compounds (0.81 < *R*^2^ < 0.99; **Figures 2D,E** and Supplementary Figure S1B), supporting a direct implication of branched lipids in the regulation of the membrane fluidity throughout the whole growth temperature intervals. Remarkably, the linear relationship observed for a given class of compounds was comparable in both strains suggesting similar adaptive mechanisms to changes in temperature. The slopes of the linear relationships appeared, however, two to four times higher for DAGEs compared to MAGEs and FAs (averaged slopes ∼-0.116, ∼-0.052 and ∼-0.032 for DAGEs, MAGEs, and FAs, respectively). These differences may be explained by the fact that DAGEs were the dominant lipids in both strains (**Figure 1**) and those preferentially involved in membrane adaptation (Supplementary Figure S2). It should also be considered that the acyl chains of AAGs present in living cells were hydrolyzed during our analytical procedure, thus probably biasing the value of the branched/linear ratio determined for MAGEs (and FAs). Among AGEs, unbranched (linear) and dimethyl branched DAGE structures seemed to play a major role in the physiological response of both *Desulfatibacillum* strains. In agreement with a regulation of the membrane fluidity, linear DAGES were formed preferentially at supra-optimal temperatures and dimethyl branched DAGEs at sub-optimal temperatures (Supplementary Figure S2), the relative proportions of both classes of compounds being strongly correlated to growth temperature (Supplementary Figure S3).

The relative proportions of branched MAGEs and DAGEs synthesized by the thermophilic strain DSM 2178 also appeared negatively correlated to growth temperature between 60 and 84°C (*R*^2^ ∼ 0.90; **Figure 2F**), but the linear relationship stopped below 60°C (**Figure 2F**). This suggests that different mechanisms of thermo-adaptation occur below 60°C to compensate changes in the physical state of membrane lipids induced by a lowering of the temperature. Similar abrupt changes in the adaptive response to temperature have been previously observed for bacterial PLFAs (Aerts et al., 1985; Reizer et al., 1985) or other cellular lipids (Grossi et al., 2000). For both mesophilic and thermophilic organisms, growth at the (inferior or superior) border of the temperature interval likely induces significant changes in enzymatic production/activity and gene regulation and leads to the formation of different protein and lipid assemblages (Hasegawa et al., 1980; Lauwers and Heinen, 1983; Aerts et al., 1985).

The fluidity of bacterial membranes can be further influenced by the location of the methyl branch(es) along the carbon chains of membrane lipids. The most central the position, the highest the membrane fluidity due to an increase of the lipid area and bilayer thickness, and a decrease of chain ordering and melting point (Knothe and Dunn, 2009; Poger et al., 2014). Methyl branches in bacterial PLFAs most commonly appear in *anteiso* and *iso* positions (Russell, 1984; Russell and Fukunaga, 1990; Denich et al., 2003; Koga, 2012) and, consequently, the relative proportions of *iso*- vs *anteiso*-PLFAs often varies with temperature. The ratio *i/a* generally increases with a rise in temperature and vice versa (Denich et al., 2003; Mansilla et al., 2004; Koga, 2012). However, branching at more central positions (e.g., C-10) is also regularly encountered (Doumenq et al., 1999; Grossi et al., 2007), and likely plays a significant role in membrane fluidity.

**FIGURE 3:**
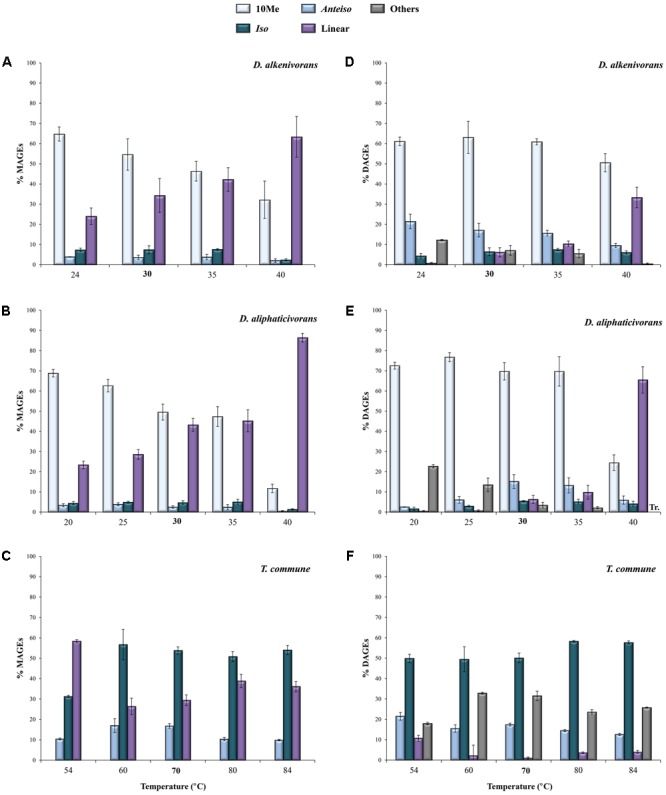
Relative abundances (% of total) of 10Me, *anteiso, iso*, and linear MAGEs **(A–C)** and DAGEs **(D–F)** released by cell hydrolysis of the mesophiles *D. alkenivorans* and *D. aliphaticivorans* and the thermophile *T. commune* grown at different temperatures. For all strains, “others” correspond to (tentatively identified) DAGEs with two methyl branches at different positions. Each data point is the mean of three independent cultures. Optimal growth temperatures are bolded.

**FIGURE 4:**
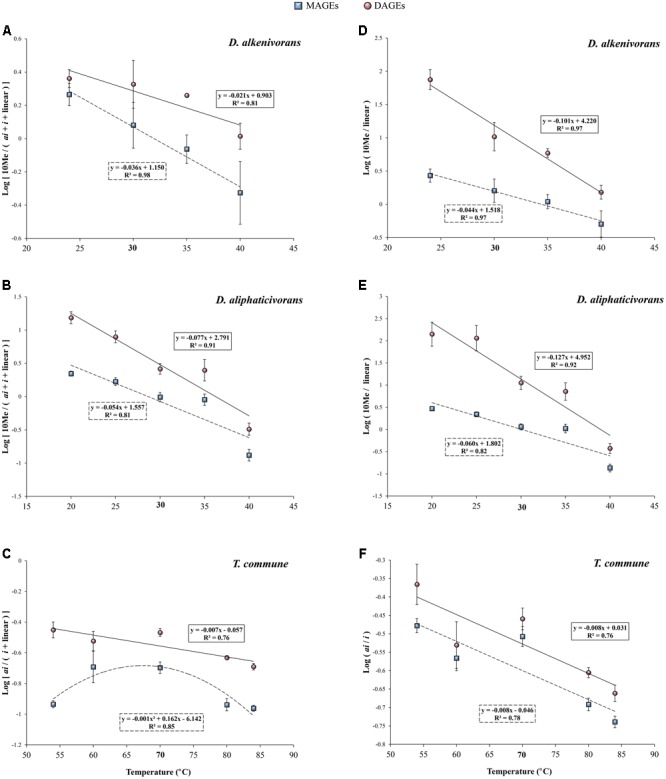
**(A,B)** Logarithm of the ratios of 10Me to *anteiso* + *iso* + linear MAGEs (squares) and monomethyl branched DAGEs (circles) in the mesophiles *D. alkenivorans* and *D. aliphaticivorans* vs growth temperature. **(C)** Logarithm of the ratio of *anteiso to iso* + linear MAGEs (squares) and DAGEs (circles) in the thermophile *T. commune* vs growth temperature. **(D,E)** Logarithm of the ratio of 10Me branched to linear MAGEs (squares) and monomethyl branched DAGEs (circles) in *D. alkenivorans* and *D. aliphaticivorans* vs growth temperature. **(F)** Logarithm of the ratio of *anteiso* to *iso* MAGEs (squares) and DAGEs (circles) in *T. commune* vs growth temperature. Each data point is the mean of three independent cultures. For *T. commune*, the ratios do not include (tentatively identified) DAGEs with two methyl branches at different positions (*ai*/*i, i*/*ai*; see Supplementary Table S1).Optimal growth temperatures are bolded.

This was actually the case for the two investigated *Desulfatibacillum* strains which biosynthesize 10Me-, *anteiso*-, and *iso*-alkyl and acyl chain(s). AGEs with a methyl branch at C-10 generally dominated the lipid composition of both strains (representing between 65 and 85% of the total hydrolyzed lipids) but were formed in lower proportion when temperature increased (**Figure 3**). The relative proportions of 10Me-branched MAGEs (**Figures 3A,B**) and DAGEs (**Figures 3D,E**) decreased progressively with increasing temperature and this was compensated by an increase of the relative proportions of unbranched (linear) homologs (**Figure 3**). The proportions of *iso*- and *anteiso*-AGEs did not seem significantly affected by temperature, being eventually slightly lower at either sides of the growth temperature interval. This is in agreement with the fact that methyl branches placed at the end of the acyl chains of PLFAs (i.e., in *anteiso* and *iso* positions) have a lower fluidizing effect than mid-chain branched carbon chains, with *iso*-PLFAs exhibiting physical properties close to unbranched-PLFAs (Poger et al., 2014). Interestingly, in both strains, the proportions of 10Me-branched MAGEs, DAGEs, and FAs relative to the sum of all other linear and branched (*anteiso* + *iso*) homologs appeared strongly correlated to temperature (*R*^2^ ≥ 0.81; **Figures 4A,B** and Supplementary Figure S1C). The linear correlations were even stronger when considering only 10Me-branched and linear structures (**Figures 4D,E** and Supplementary Figure S1D). This attested for a major implication of 10Me and linear alkyl (and acyl) chains in the regulation of membrane fluidity by the two investigated mesophilic strains in response to changing temperature.

Unlike *Desulfatibacillum* strains, *T. commune* did not synthesize 10Me-branched lipids and only produced *iso*- and *anteiso*-structures in addition to unbranched alkyl and acyl chains. As commonly observed in thermophilic bacteria (Koga, 2012), *iso*-alkyl chains were dominant in *T. commune*, representing ca. 50–60% of total hydrolyzed lipids (**Figures 3C,F**) and 71% of the total AGEs. This proportion remained relatively constant between 84 and 60°C, but dropped to ca. 55% at 54°C where it was compensated by a doubling (from <10% to ca. 20%) of linear alkyl chains (**Figures 3C,F** and Supplementary Figure S2). *Anteiso*-alkyl chains represented ca. one-fourth of total alkyl chains under optimal and sub-optimal conditions of temperature (i.e., between 50 and 70°C) but were less present (ca. 15%) at supra-optimal growth temperatures (**Figure 3C**) in accordance with their fluidizing properties and probable involvement in membrane homeoviscous adaptation. The proportion of *anteiso*-DAGEs relative to *iso*- and linear homologs [i.e., ai/(*i* + linear) DAGEs] appeared linearly correlated with temperature across the full range of temperature tested (**Figure 4C**). This relationship was not systematically verified for the least abundant lipid compounds, MAGEs and FAs, for which *anteiso*-structures were less represented under both sub- and supra-optimal conditions of temperature (**Figures 4C,F** and Supplementary Figures S1E,F).

##### Position of the alkyl chain in MAGEs

**FIGURE 5:**
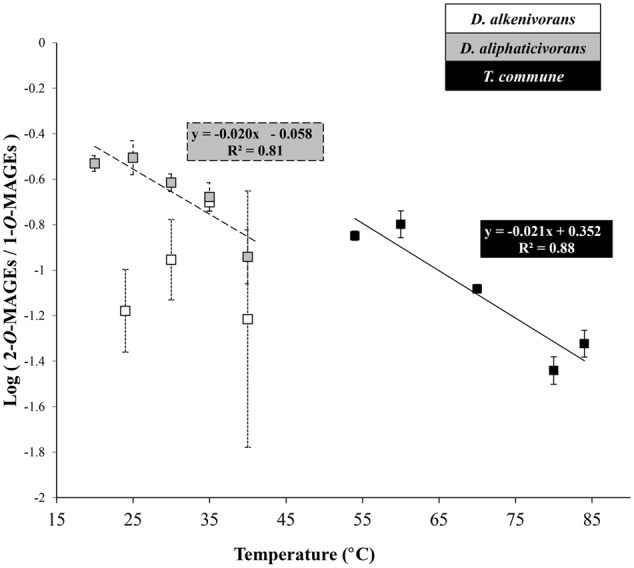
Logarithm of the ratio of 2-*O*- to 1-*O*-MAGEs produced by the mesophiles *D. alkenivorans* and *D. aliphaticivorans* and the thermophile *T. commune* as a function of growth temperature. Each data point is the mean of three independent cultures.

In addition to modifications of the chain length and branching pattern of the alkyl and acyl chains of membrane phospholipids, changes of the growth temperature also affected the relative proportions of *sn*-1 and *sn*-2 MAGEs synthesized by the three strains (**Figure 5**). In the mesophile *D. aliphaticivorans* and the thermophile *T. commune*, the proportion of 2-*O*-MAGEs relative to 1-*O*-MAGEs gradually decreased with increasing temperature, showing a strong linear correlation with growth temperature (**Figure 5**). Remarkably, the slope of the linear regression describing the correlation was comparable for both strains, suggesting a similar mechanism of regulation of the proportions of *sn*-2 vs *sn*-1 MAGEs regardless of temperature growth preferences. It should be noted, however, that a similar correlation was not observed for the second mesophilic *D. alkenivorans* for which no clear trend was observed (**Figure 5**). To our knowledge, the report of a modification of the relative proportions of *sn*-1*/sn*-2 MAGEs in response to changing environmental conditions is unprecedented. The possibility that changes in temperature partially inhibited the (yet unknown) mechanisms responsible for the formation of one either ether linkage is unlikely since the proportion of DAGEs relative to MAGEs appeared unchanged across the full range of temperature tested (**Figures 1D–F**). Differences in the physicochemical properties of 1-*O*- vs 2-*O*-MAGES are unknown and it is presently difficult to attest that *sn*-1 ether lipids have a greater fluidizing effect than *sn*-2 homologs. The present observations suggest, however, that the regulation of the proportion of 2-*O*-/1-*O*-ether lipids may constitute an additional mechanism employed by some alkylglycerol-synthesizing bacteria to control the fluidity (phase transition temperature) of their membranes and cope with shifts in growth temperature. Further studies with other AGE-producers would be needed to determine if such an adaptive trait occurs more widely in Bacteria.

### Potential Usefulness of Alkyl Glycerols as Environmental Indicators

Recent studies have investigated the possibility that distinct distributions of MAGEs or DAGEs in natural settings may reflect an adaptive behavior of microorganisms to varying environmental conditions. The DAGE composition of diverse geothermal sinters in New Zealand was shown to depend on the pH (from 2.1 to 9.0) and temperature (from 68 to 98°C) of the site in addition to being influenced by differences in the microbial community (Kaur et al., 2015). Disentangling the individual effect of one either physicochemical parameter in such complex geothermal settings appeared, however, difficult, and correlations between the ACL of DAGEs and temperature or pH could only be obtained by removing the extreme pH or temperature samples. The chain length and the degree of unsaturation of 1-*O*-MAGEs present in suspended particulate organic matter from the eastern South Atlantic and the Southern Ocean were also suspected to be linked to seawater temperature (Hernandez-Sanchez et al., 2014), but no clear correlation between MAGE structures and temperature was highlighted. Difficulties in using modifications of the ACL of AGEs as an indicator of temperature changes further relies on the non-systematic (and sometimes paradoxical) response observed from one bacterial strain to another (e.g., **Figures 2A–C** and discussion above). However, the ACL of AGEs in bacteria appears related to the optimal growth temperature (**Figures 2A–C**; Jahnke et al., 2001), possibly allowing the psychrophilic, mesophilic, thermophilic, or hyperthermophilic character of bacteria to be distinguished based on this averaged structural characteristic. On the other hand, the use of the degree of unsaturation of AGEs to trace temperature changes in the environment seems complicated because unsaturated AGEs are less common and less resistant to diagenesis than their saturated counterparts.

The strong correlations observed between modifications of the level of methyl branching and/or the specific methylation pattern of AGEs and temperature (**Figures 2, 4** and Supplementary Figure S3) allow envisaging the use of AGEs as potential indicators of temperature changes in natural environments. Indeed, such modifications are in good agreement with a regulation of the membrane fluidity regardless of the growth temperature optimum/range, and with previous observations made with PLFAs (Denich et al., 2003; Poger et al., 2014). Moreover, several ratios of branched to linear structures of MAGEs and DAGEs appear correlated to temperature (**Figures 2, 4** and Supplementary Figure S3), which may facilitate the choice of an adequate ratio to use depending on the type of environment considered and diversity of AGE encountered. The ratios of (total) branched to linear MAGEs or DAGEs (**Figures 2D–F**) and of (total) dimethyl branched to linear DAGEs (Supplementary Figure S3) are likely the easiest ratios to determine in a given sample, as they do not require the precise structural identification of the compounds. Those latter can simply be distinguished based on their molecular weights and chromatographic retention times. Whenever possible, subtler and specific distributional variations in methyl branched MAGEs and DAGEs can also be investigated. Specifically, the ratios of 10Me to linear and of *anteiso* to *iso* MAGEs and DAGEs (**Figures 4D–F**) may constitute powerful indicators of temperature changes in temperate and (hyper)thermophilic environments, respectively. Noteworthy is the fact that adaptation to changes in pH also potentially influences the methylation pattern of AGEs. A significant negative correlation between the *iso* to *anteiso* ratio of 1-*O*-MAGEs and soil pH was observed during the study of Chinese soils, but no relationship between the two parameters was evidenced for lake sediments (Yang et al., 2015). An effect of pH on branching of MAGEs and DAGEs may thus be restricted to environments characterized by large intervals of pH, such as soils (Yang et al., 2015) or extreme environments (Kaur et al., 2015), and eventually be used to distinguish different type of paleoenvironments (Wang and Xu, 2016). Additional culture experiments focusing on the influence of pH on the AGE composition of bacteria undoubtedly deserve investigation.

Conclusively, the strong correlations observed between specific structural features of MAGEs and DAGEs and the growth temperature of marine bacteria might allow the use of these thermostable lipids as a new tool for tracing temperature changes in aquatic ecosystems back in time, in a way comparable to other molecular proxies (Eglinton and Eglinton, 2008). Studies on the distributional variations in AGEs in (ancient) marine sedimentary settings characterized by contrasting temperatures should help supporting this latter hypothesis.

## Author Contributions

AV-L performed the analyses, interpreted the data, drew figures and tables, and wrote the first draft of the manuscript. IM performed SRB cultivation. VG and CC-L conceived the project, helped interpreting the data and revised the whole manuscript.

## Conflict of Interest Statement

The authors declare that the research was conducted in the absence of any commercial or financial relationships that could be construed as a potential conflict of interest.
