# Supplementary material for: Temperature-Dependent Alkyl Glycerol Ether Lipid Composition of Mesophilic and Thermophilic Sulfate-Reducing Bacteria
Source: Front Microbiol. 2017 Aug 9;8:1532. doi: 10.3389/fmicb.2017.01532 (PMC5552659; doi:10.3389/fmicb.2017.01532)
Supplement: Supplementary file 1 [file Table_1.DOCX]

**Table S1.** Relative proportions (% of total lipids) of individual fatty acids (FAs), monoalkyl glycerols (MAGEs) and dialkyl glycerols (DAGEs) released by acid hydrolysis of cells of *T. commune* DSM 2178 grown at 70°C (optimal growth temperature). Relative abundances and standard deviations were calculated on the basis of three independent cultures. tr., traces (< 0.1%). Major compounds (> 3%) are highlighted in bold.

| FAs | |  | MAGEs | |  | DAGEs | |
| --- | --- | --- | --- | --- | --- | --- | --- |
|  |  |  |  |  |  |  |  |
| *i*C_14_ | tr |  | 1-*O*-*i*C_15_ | tr |  | 1,2-*O*-*i*C_15_/*i*C_16_ | tr |
| *n*C_14_ | tr |  | 1-*O*-*ai*C_15_ | tr |  | 1,2-*O*-*ai*C_15_/*i*C_16_ ***** | 0.1 *± 0.0* |
| *i*C_15_ | tr |  | 2-*O*-*i*C_16_ | 0.3 *± 0.0* |  | **1,2-*O*-*i*C_16_/*i*C_16_** | **3.8 *± 0.2*** |
| *ai*C_15_ | tr |  | 1-*O*-*i*C_16_ | 0.6 *± 0.0* |  | 1,2-*O*-*i*C_15_/*ai*C_17_ ***** | 0.2 *± 0.0* |
| ***i*C_16_** | **5.0 *± 0.4*** |  | 2-*O*-*n*C_16_ | tr |  | 1,2-*O*-*ai*C_15_/*ai*C_17_ | 0.1 *± 0.0* |
| *n*C_16_ | 0.7 ± 0.0 |  | 1-*O*-*n*C_16_ | 0.3 *± 0.0* |  | 1,2-*O*-*n*C_16_/*i*C_16_ ***** | 1.5 *± 0.0* |
| *i*C_17_ | 1.0 *± 0.1* |  | 2-*O*-*i*C_17_ | tr |  | 1,2-*O*-*n*C_16_/*n*C_16_ | 0.1 *± 0.0* |
| ***ai*C_17_** | **5.8 *± 0.3*** |  | 1-*O*-*i*C_17_ | 0.5 *± 0.0* |  | 1,2-*O*-*i*C_17_/*i*C_16_ | 1.6 *± 0.1* |
| *n*C_17_ | tr |  | 2-*O*-*ai*C_17_ | 0.3 *± 0.0* |  | 1,2-*O*-*i*C_16_/*i*C_17_ | 0.7 *± 0.1* |
| *i*C_18_ | 2.7 *± 0.2* |  | 1-*O*-*ai*C_17_ | 1.0 *± 0.1* |  | **1,2-*O*-*i*C_16_/*ai*C_17_** ***** | **3.8 *± 0.5*** |
| *n*C_18_ | 1.8 *± 0.3* |  | 1-*O*-*n*C_17_ | tr |  | **1,2-*O*-*ai*C_17_/*i*C_16_** ***** | **3.8 *± 0.5*** |
| *i*C_19_ | tr |  | 2-*O*-*i*C_18_ | tr |  | 1,2-*O*-*n*C_16_/*i*C_17_ ***** | 0.1 *± 0.1* |
| *ai*C_19_ | 0.2 *± 0.0* |  | **1-*O*-*i*C_18_** | **3.8 *± 0.3*** |  | 1,2-*O*-*i*C_17_/*n*C_16_ ***** | 0.1 *± 0.1* |
| *n*C_19_ | tr |  | 2-*O*-*n*C_18_ | tr |  | 1,2-*O*-*n*C_16_/*ai*C_17_ ***** | 1.5 *± 0.4* |
| *i*C_20_ | tr |  | 1-*O*-*n*C_18_ | 2.6 *± 0.5* |  | **1,2-*O*-*i*C_18_/*i*C_16_** | **15.9 *± 1.0*** |
| *n*C_20_ | tr |  | 2-*O*-*i*C_19_ | tr |  | 1,2-*O*-*i*C_17_/*i*C_17_ | 0.3 *± 0.0* |
|  |  |  | 1-*O*-*i*C_19_ | tr |  | **1,2-*O*-*ai*C_17_/*i*C_17_** ***** | **3.2 *± 0.1*** |
|  |  |  | 1-*O*-*ai*C_19_ | 0.3 *± 0.1* |  | **1,2-*O*-*ai*C_17_/*ai*C_17_** | **4.3 *± 0.7*** |
|  |  |  | 1-*O*-*n*C_19_ | tr |  | **1,2-*O*-*n*C_18_/*i*C_16_** ***** | **8.7 *± 0.7*** |
|  |  |  | 1-*O*-*i*C_20_ | tr |  | 1,2-*O*-*n*C_18_/*n*C_16_ | 0.6 *± 0.4* |
|  |  |  | 1-*O*-*n*C_20_ | tr |  | 1,2-*O*-*i*C_18_/*i*C_17_ | 1.4 *± 0.4* |
|  |  |  |  |  |  | 1,2-O-*i*C_17_/*i*C_18_ | 0.1 *± 0.0* |
|  |  |  |  |  |  | **1,2-*O*-*i*C_18_/*ai*C_17_** ***** | **11.6 *± 1.1*** |
|  |  |  |  |  |  | 1,2-*O*-*n*C_18_/*i*C_17_ ***** | 0.9 *± 0.0* |
|  |  |  |  |  |  | **1,2-*O*-*n*C_18_/*ai*C_17_** ***** | **6.3 *± 0.3*** |
|  |  |  |  |  |  | 1,2-*O*-*n*C_18_/*n*C_17_ | tr |
|  |  |  |  |  |  | 1,2-*O*-*i*C_18_/*i*C_18_ | 0.8 *± 0.1* |
|  |  |  |  |  |  | 1,2-*O*-*i*C_19_/*ai*C_17_ ***** | 0.3 *± 0.1* |
|  |  |  |  |  |  | 1,2-*O*-*ai*C_19_/*ai*C_17_ | 0.4 *± 0.0* |
|  |  |  |  |  |  | 1,2-*O*-*n*C_18_/*i*C_18_ ***** | 0.5 *± 0.2* |
|  |  |  |  |  |  | 1,2-*O*-*n*C_18_/*n*C_18_ | 0.1 *± 0.0* |

***** Tentative assignment of the position of the branched alkyl chain(s) on the glycerol moiety (1-*O*- *vs* 2-*O*-)
