# Supplementary material for: Temperature-Dependent Alkyl Glycerol Ether Lipid Composition of Mesophilic and Thermophilic Sulfate-Reducing Bacteria
Source: Front Microbiol. 2017 Aug 9;8:1532. doi: 10.3389/fmicb.2017.01532 (PMC5552659; doi:10.3389/fmicb.2017.01532)
Supplement: Supplementary file 2 [file Data_Sheet_1.DOCX]

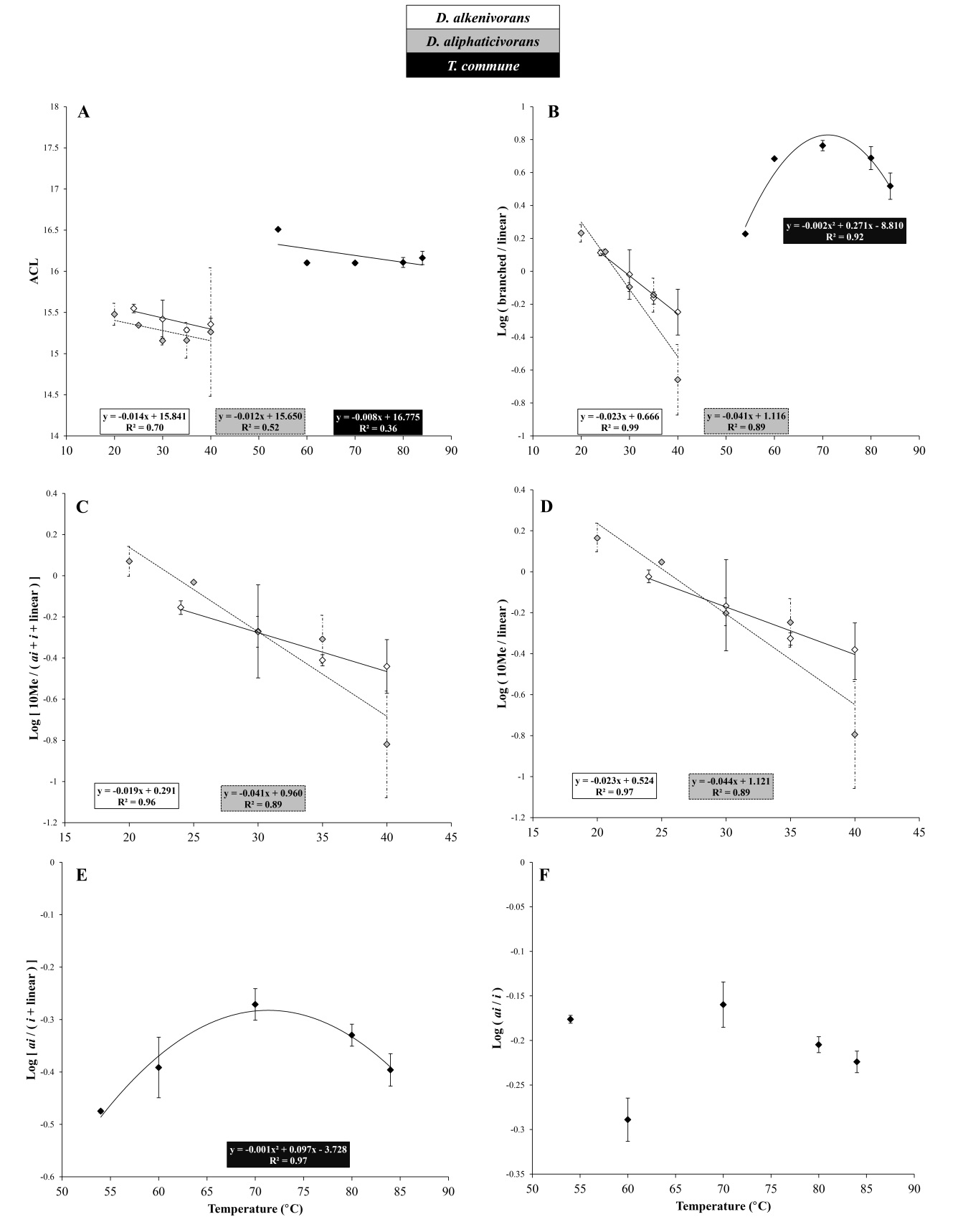


**Fig. S1.** Weighted ACL (**A**) and logarithm of the ratio of branched to linear structures (**B**) of FAs produced by two *Desulfatibacillum* strains and the thermophile *T. commune* as a function of growth temperature. Logarithm of the ratios of 10-Me to a*nteiso*+*iso*+linear FAs (**C**) and of 10-Me to linear FAs (**D**) in *Desulfatibacillum* strains versus growth temperature. Logarithm of the ratios of *anteiso* to *iso*+linear FAs (**E**) of *anteiso* to *iso* FAs (**F**) in *T. commune* versus growth temperature. Each data point is the mean of three independent cultures.
