# Supplementary material for: Temperature-Dependent Alkyl Glycerol Ether Lipid Composition of Mesophilic and Thermophilic Sulfate-Reducing Bacteria
Source: Front Microbiol. 2017 Aug 9;8:1532. doi: 10.3389/fmicb.2017.01532 (PMC5552659; doi:10.3389/fmicb.2017.01532)
Supplement: Supplementary file 3 [file Data_Sheet_2.DOCX]

**
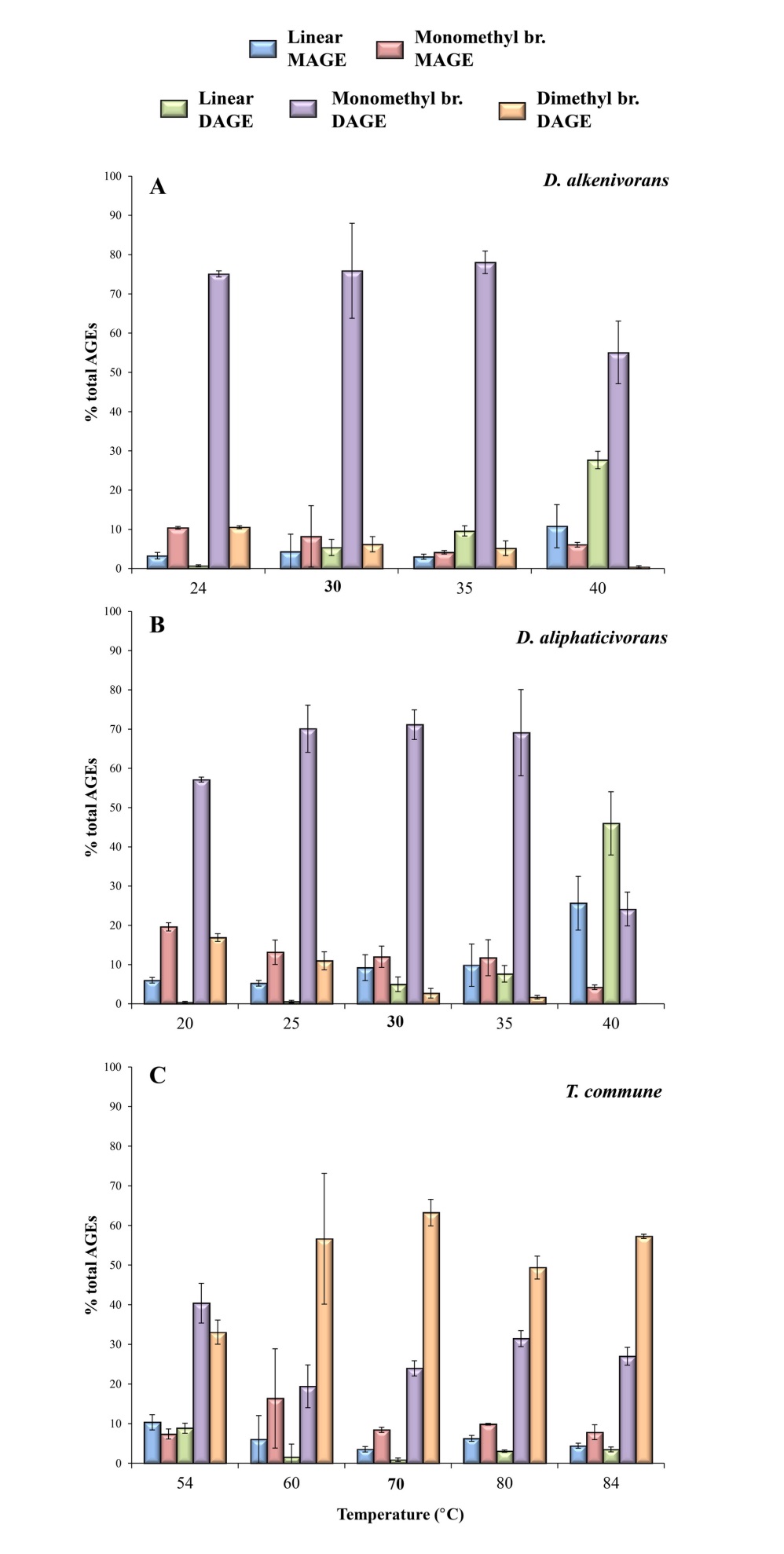
**

**Fig. S2.** Relative proportions (mean of three independent cultures) of linear and methyl branched MAGEs and DAGEs (% of total AGEs) in two mesophilic *Desulfatibacillum* strains and the thermophile *T. commune* grown at different temperatures. Optimal growth temperatures are bolded.
