# Supplementary material for: Temperature-Dependent Alkyl Glycerol Ether Lipid Composition of Mesophilic and Thermophilic Sulfate-Reducing Bacteria
Source: Front Microbiol. 2017 Aug 9;8:1532. doi: 10.3389/fmicb.2017.01532 (PMC5552659; doi:10.3389/fmicb.2017.01532)
Supplement: Supplementary file 4 [file Data_Sheet_3.DOCX]

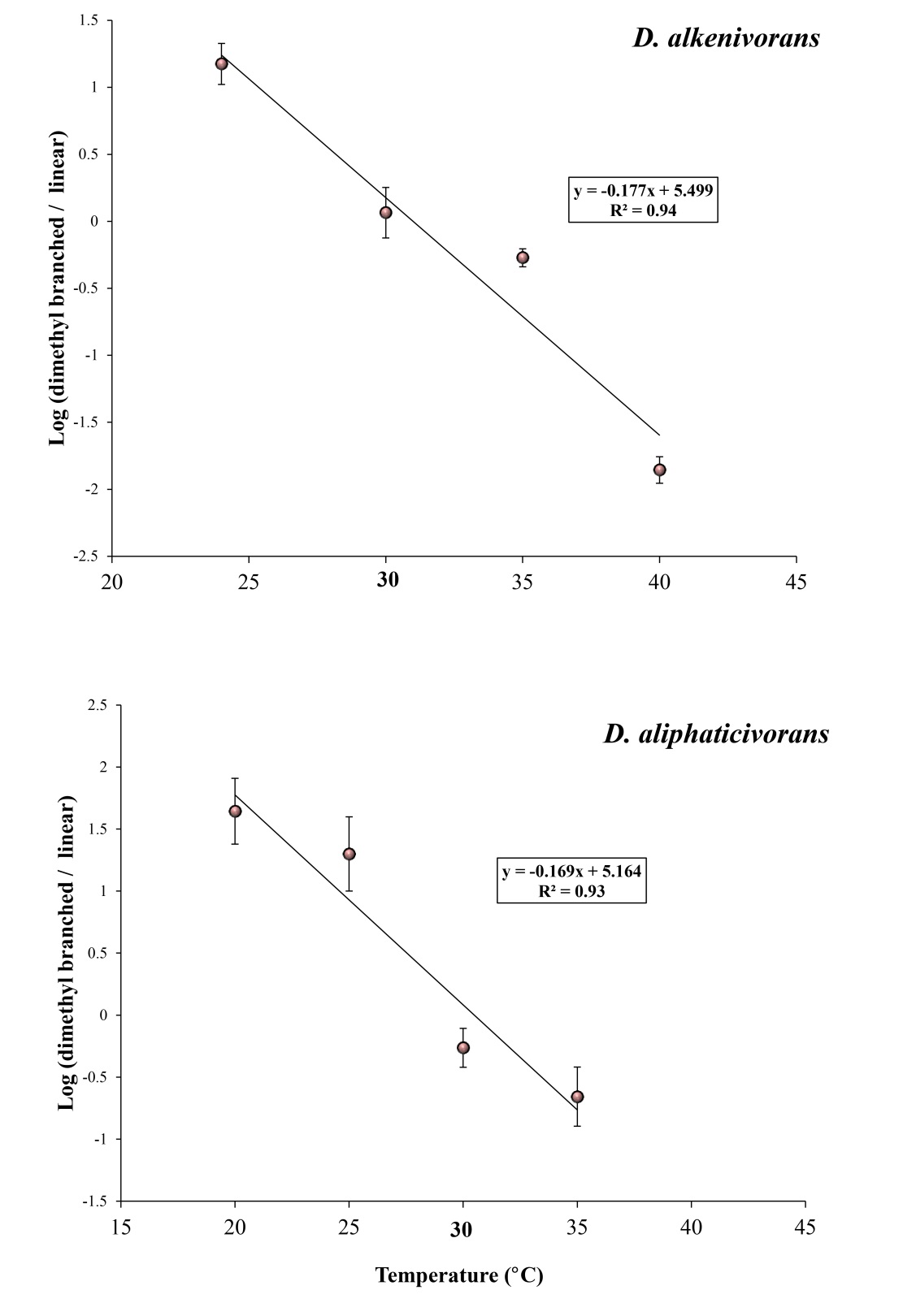


**Fig. S3.** Logarithm of the ratio of all DAGEs with two branched alkyl chains to linear DAGEs in *Desulfatibacillum* strains versus growth temperature. Each data point is the mean of three independent cultures. Optimal growth temperatures are bolded.
